# Current Approaches Toward Quantitative Mapping of the Interactome

**DOI:** 10.3389/fgene.2016.00074

**Published:** 2016-05-04

**Authors:** Alexander Buntru, Philipp Trepte, Konrad Klockmeier, Sigrid Schnoegl, Erich E. Wanker

**Affiliations:** Max Delbrueck Center for Molecular MedicineBerlin, Germany

**Keywords:** PPI analysis, FRET, DULIP, FCCS, PLA, Interactome Mapping, BiFC, LUMIER, BRET, Quantification of protein-protein interactions

## Abstract

Protein–protein interactions (PPIs) play a key role in many, if not all, cellular processes. Disease is often caused by perturbation of PPIs, as recently indicated by studies of missense mutations. To understand the associations of proteins and to unravel the global picture of PPIs in the cell, different experimental detection techniques for PPIs have been established. Genetic and biochemical methods such as the yeast two-hybrid system or affinity purification-based approaches are well suited to high-throughput, proteome-wide screening and are mainly used to obtain qualitative results. However, they have been criticized for not reflecting the cellular situation or the dynamic nature of PPIs. In this review, we provide an overview of various genetic methods that go beyond qualitative detection and allow quantitative measuring of PPIs in mammalian cells, such as dual luminescence-based co-immunoprecipitation, Förster resonance energy transfer or luminescence-based mammalian interactome mapping with bait control. We discuss the strengths and weaknesses of different techniques and their potential applications in biomedical research.

## Introduction

Physical interactions between proteins are crucial to most biological processes. Hence, major efforts have been made to systematically identify protein–protein interactions (PPIs) using the yeast two-hybrid (Y2H) system and affinity purification–mass spectrometry (AP/MS) approaches (Stelzl et al., 2005; Yu et al., 2008; Guruharsha et al., 2011). However, these methods are mainly suited for providing qualitative data, especially at the large scale. For a more comprehensive functional description of interactions, additional information is required. Knowledge of interaction strength, e.g., is of particular importance. It informs us of binding affinities and lifetimes of protein complexes, which are critical for the dynamic regulation of cellular systems (Perkins et al., 2010; Hieb et al., 2012). In summary, a better understanding of complex cellular processes not only requires knowledge of which proteins interact but also of the characteristics of interactions. To obtain such insight, quantitative experimental techniques for the detection of PPIs in mammalian cells have moved into focus (Hieb et al., 2012; Chen et al., 2015). These include biochemical methods such as quantitative affinity-purification and mass spectrometry (qAP–MS; Hosp et al., 2015) or genetic methods such as using luminescence-based mammalian interactome mapping with bait control (LUMIER with BACON; Taipale et al., 2014). Using qAP–MS, e.g., the association of proteins with neurodegenerative disease proteins such as amyloid precursor protein (APP), presenilin-1 and ataxin-1 (ATXN-1) have been quantitatively analyzed and the effects of disease-causing mutations on PPIs have been systematically assessed in pull-down assays (Hosp et al., 2015). The quantitative investigation of PPIs using LUMIER with BACON revealed a comprehensive Hsp90–client interaction network, which provided insight into previously unknown organization principles of functional chaperone modules in mammalian cells (Taipale et al., 2014).

A recent study suggests that about 60% of disease-causing mutations in proteins influence their association with other proteins. It was estimated that half of those mutations leads to a complete loss of protein interactions while the other half only perturbs a particular subset of interactions (Sahni et al., 2015). A pathological poly-glutamine expansion in ATXN-1, causally related to spinocerebellar ataxia type 1 (SCA1), e.g., was found to induce binding of the protein to RBM17 rather than CiC, thereby promoting disease (Lim et al., 2008). To detect such changes in affinity and to map how interaction profiles of individual proteins are changed through mutations, methods that allow quantitative PPI analysis are urgently needed.

However, the available methodologies do not yet permit a full quantitative assessment of PPIs at the cellular level. Current methods to study binary PPIs in mammalian cells can broadly be classified in two groups. Assays like bimolecular fluorescence complementation (BiFC), bimolecular luminescence complementation (BiLC) and proximity ligation assay (PLA) yield a quantitative readout without allowing conclusions about interaction strengths, while assays like Förster resonance energy transfer (FRET), bioluminescence resonance energy transfer (BRET), fluorescence cross-correlation spectroscopy (FCCS), dual luminescence-based co-immunoprecipitation (DULIP) and LUMIER with BACON provide a quantitative readout that can be used to determine binding strengths. In this paper, we will review recent developments in quantitative PPI detection technologies and provide an overview of relevant applications of these methods in biomedical research. We focus on genetic approaches in mammalian cells, as mass spectrometry-based methods have been recently reviewed elsewhere (Meyer and Selbach, 2015). Protein microarrays also provide important insights on PPIs and can provide quantitative readouts (MacBeath and Schreiber, 2000; Jones et al., 2006). They also have been reviewed elsewhere and will not be discussed here (Wolf-Yadlin et al., 2009).

An overview of the discussed methods and their capabilities is provided in **Table 1**.

**Table 1:** Overview of capabilities of binary PPI detection methods in mammalian cells.

Method	FCCS	BiFC/BiLC	PLA	FRET	BRET	LUMIER	DULIP
PPI assay principle	Co-migration of proteins	Protein fragment complementation	Proximity-based ligation of oligonucleotides	Förster resonance energy transfer	Bioluminescence resonance energy transfer	Co-immunoprecipitation	Co-immunoprecipitation
PPI read-out	Fluorescence	Fluorescence/luminescence	Fluorescence/luminescence	Fluorescence	Luminescence	Luminescence	Luminescence
Quantification of binding strengths	Yes	No	No	Yes	Yes	Yes	Yes
Detection of PPIs in intact cells	Yes	Yes	No	Yes	Yes	No	No
Detection of PPIs in lysed cells	Yes	Yes	Yes	Yes	Yes	Yes	Yes
Subcellular localization of PPIs	No	Yes	Yes	Yes	Yes	No	No
Detection of endogenous PPIs	No	No	Yes	No	No	No	No
Expression of tagged proteins required	Yes	Yes	No	Yes	Yes	Yes	Yes
Throughput of PPI detection assay	Medium	High	Low	High	High	High	High

*Several methods can be used for high-throughput interaction mapping. Fluorescence and luminescence play a prominent role in current approaches.*

## Fluorescence Cross-Correlation Spectroscopy

Fluorescence correlation spectroscopy (FCS) was described for the first time over 40 years ago (Magde et al., 1974; Macháň, 2014). It was developed to measure chemical reaction rates and diffusion coefficients by analyzing the thermodynamic fluctuations in the fluorescence intensity of a system. FCS is now a well-established biophysical method, which in combination with confocal microscopy is routinely used to obtain quantitative information about the abundance of fluorescently tagged proteins in living cells (Macháň, 2014). Through the expansion of the method to dual-color FCCS it became possible to quantify interactions of labeled proteins *in vivo* under physiological conditions (Schwille et al., 1997).

**FIGURE 1:**
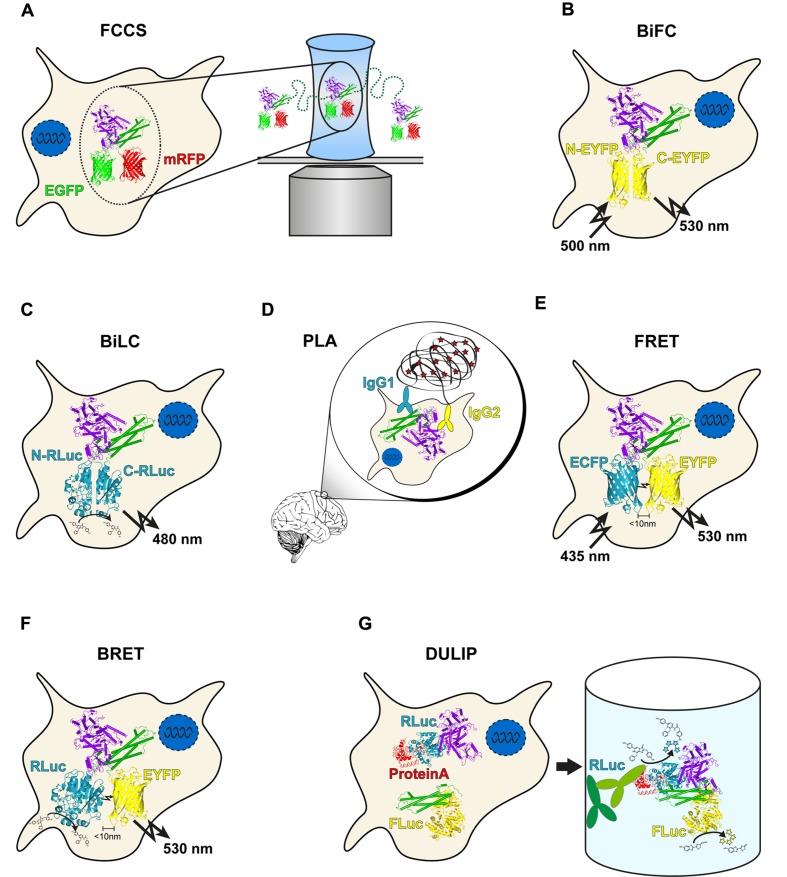
**Overview of genetic protein–protein interaction (PPI) methods. (A)** In Fluorescence cross-correlation spectroscopy (FCCS) measurements, co-migration of two fluorescently labeled molecules through a focal volume is quantified. **(B)** bimolecular fluorescence complementation (BiFC) utilizes two non-fluorescent fragments of EGFP or a variant. Upon interaction of the two labeled proteins, the fragments can reassociate, resulting in fluorescence. **(C)** The principle of bimolecular luminescence complementation (BiLC) is similar to BiFC but is based on two fragments of a luciferase. In contrast to BiFC, the reassociation is reversible. **(D)** Close proximity of two DNA oligomer-labeled antibodies allows circularization of two additional oligomers after hybridization. The product is amplified in a rolling circle reaction and subsequently detected with fluorescently labeled probes. **(E)** During Förster resonance energy transfer (FRET), energy is transferred non-radiatively from an excited donor molecule to an acceptor molecule. In case the acceptor is also a fluorophore, the transmitted energy is emitted at a longer wavelength (the so called sensitized emission). **(F)** bioluminescence resonance energy transfer (BRET) is similar to FRET with the difference that a luciferase serves as a donor molecule. **(G)** In dual luminescence-based co-immunoprecipitation (DULIP) assays, two proteins of interest are fused to firefly or *Renilla* luciferase, respectively. An additional PA-tag allows precipitation of the bait protein from the lysate. If an interaction occurs, co-precipitation of the prey protein is indicated by luminescence arising from the firefly luciferase.

Fluorescence cross-correlation spectroscopy allows the measurement of protein mobility, concentration and interactions by exploiting the temporal fluorescence fluctuations of two fluorescently labeled particles under a confocal laser scanning microscope diffusing through a minute focal volume (**Figure 1A**). As a distinct number of fluorescently labeled molecules diffuse through the focal volume (Haustein, 2014), the fluorescence signals fluctuate in a manner dependent on the mobility and concentration of the investigated proteins. An autocorrelation function of the fluctuating fluorescence signals provides the diffusion coefficients and concentrations of molecules. Importantly, FCCS utilizes two spectrally different fluorophores, e. g., monomerized green or red fluorescent proteins, to label a pair of proteins (Bacia et al., 2006). If the differently labeled proteins are associated with each other, they pass through the effective volume in a synchronized way. This causes simultaneous fluctuation of their fluorescence signals leading to an increase in the amplitude of the cross-correlation function, allowing the determination of *in vivo* interaction strengths for proteins of interest (Boeke et al., 2014). However, co-migration does not fully prove a direct binary interaction of two-labeled molecules. Thus, validation with other methods that are more stringent in this regard is necessary (Shi et al., 2009).

Quantitative *in vivo* FCCS analysis, e.g., revealed binding strengths for PPIs involved in the extracellular signal-regulated kinase/mitogen-activated protein kinase (ERK/MAPK) pathway (Sadaie et al., 2014). The generated quantitative data was utilized to perform computer-assisted simulations to model the ERK-/MAPK-signaling cascade, uncovering that Shc binding to EGFR is critical for the regulation of the pathway. Similarly, systematic FCCS studies of 41 PPIs revealed important information about the regulation of clathrin-mediated endocytosis in yeast (Boeke et al., 2014). Through the *in vivo* measurement of interaction strengths for selected interactions likely to be involved in endocytosis the protein Ede1 was discovered as a crucial scaffold for the organization of this process. These results highlight the application power of FCCS for quantitative detection of PPIs in cells and show that quantitative PPI information improves our current understanding of signal transduction pathways. Through the systematic application of FCCS it seems feasible that comprehensive, quantitative interactome maps can be generated in the future.

## Bimolecular Complementation Methods: Protein-Fragment Complementation Assay (Pca), BiFc, And BiLc

Protein-fragment complementation assays have been utilized for a long time to detect PPIs in yeast or mammalian cells (Johnsson and Varshavsky, 1994; Kerppola, 2006). PCAs are classical reporter assays, in which a fluorescent protein or enzyme, e.g., is split in two and the parts are then fused to the N- or C-terminal end of the potential interactors. If the proteins of interest interact, the fragments unite, emitting measurable fluorescence or displaying quantifiable enzymatic activity. Different PCA variants have been used successfully in small- or proteome-scale applications to detect PPIs (Tarassov et al., 2008; Sung et al., 2013; Petschnigg et al., 2014).

One of the most commonly used PCA variants is the BiFC assay, which is based on the reconstitution of a fluorescent protein such as the green fluorescent protein (GFP) (Kerppola, 2008; Kodama and Hu, 2012). In an application of BiFC, an N-terminal GFP fragment (NGFP) containing the first 157 amino acids and a C-terminal GFP fragment (CGFP) fragment containing 81 terminal amino acids were fused to peptides that are known to assemble into antiparallel leucine zippers (Ghosh et al., 2000). The interaction of the peptides led to the reconstitution of functional GFP molecules that exhibited a single excitation maximum at 475 nm and an emission maximum at 506 nm. Today, multiple BiFC assays with many different split fluorescent proteins (FPs) are available for application, including ECFP, EGFP, EYFP (**Figure 1B**), Venus, Citrine, Cerulean, or mCherry [reviewed in Miller et al., 2015]. However, all PPI detection assays with split-FP variants suffer from spontaneous self-assembly of the utilized fragments, which results in relatively high background fluorescence in cells. To overcome this limitation, variants of the Venus-based BiFC PPI detection system with an improved signal-to-noise ratio were developed (Kodama and Hu, 2010). Another disadvantage of most if not all currently available BiFC methods is that split–FP fusions form irreversible protein complexes *in vitro* and *in vivo*, which can lead to false-positive results. Also, they only allow measuring the association of protein complexes but not their dissociation (Ciruela et al., 2010).

A related PCA is the BiLC assay, which uses luciferases rather than fluorescent proteins (**Figure 1C**). As for BiFC, several variants of the method are available that utilize different luciferases such as firefly (Paulmurugan et al., 2002), *Renilla* (Paulmurugan and Gambhir, 2003), *Gaussia* (Remy and Michnick, 2006), or NanoLuc^®^ (Dixon et al., 2015). Importantly, the reconstitution of the luciferase fragments is reversible in these assays, allowing the detection of both association and dissociation of fusion proteins in living cells in real-time (Remy and Michnick, 2006). Compared to BiFC, BiLC assays offer a higher signal-to-noise ratio, which is very advantageous for the large-scale detection of PPIs in cells. BiLC assays were also utilized to study the localization of PPIs in cells (Kaihara et al., 2003). However, due to the relatively low number of emitted photons this can be a very challenging task (Kato, 2012).

The power of BiFC-based PPI detection methods lies in their ability to identify weak or transient interactions in cells (Miller et al., 2015). This is because fusion proteins are stabilized in complementation assays and fluorescent signals are only observed when the tagged fusions interact directly. The relatively weak interaction between the SH3 domain of c-Abl and the poly-proline peptide p41 (*K*_d_ = 1.5 μM), e.g., could be readily detected in intact cells using a YFP-based BiFC assay (Morell et al., 2007). Recently, a recombinase enhanced bimolecular luciferase complementation (ReBiL) platform was established that allows the detection of low-affinity PPIs in living cells. It enabled the discovery of the interaction between the E3 ubiquitin ligase FANCL and the ubiquitin-conjugating enzyme UBE2T (*K*_d_ = 0.454 μM), two key players in DNA repair processes (Li et al., 2014).

BiFC and BiLC allow rapid, sensitive investigation of PPIs in cells with a quantitative data output both in focused experiments as well as on the proteome scale (Sung and Huh, 2010). To assess binding affinities of interacting proteins in cells, however, both unbound and bound protein molecules would need to be quantified. This is not possible with BiFC or BiLC assays because only interacting fusion proteins show fluorescence or luminescence complementation (**Figures 1B,C**). Finally, it is important to note that the lack of information on unbound FPs in BiFC assays may lead to false positive as well as false negative results in systematic PPI screenings, simply because the expected bait and prey fusions may not be properly expressed in cells.

## Proximity Ligation Assays

The proximity ligation assay utilizes antibodies to which short single-stranded DNA oligonucleotides, often termed PLA probes, have been attached (Fredriksson et al., 2002; Söderberg et al., 2006; Weibrecht et al., 2010). When bound to two proteins that are in close proximity in biological systems (distance < 30 nm), these antibody–DNA probes facilitate the ligation of additional DNA molecules by ligases and subsequent amplification by polymerase chain reaction or a rolling circle mechanism. The amplified DNA molecules function as templates for the binding of fluorescently labeled oligonucleotide probes that act as surrogate markers for interacting proteins (**Figure 1D**). The dual recognition by PLA probes required for the formation of DNA reporter molecules decreases non-specific signals because only ligated reporters are amplified (Weibrecht et al., 2010).

Proximity ligation assays have the advantage over methods like BiFC or FCCS that associations between proteins can be identified and quantified without additional tags. The only requirement is the availability of specific, high-affinity antibodies against the proteins of interest that can be modified with DNA oligonucleotides. In the last 10 years, multiple variants of PLAs have been developed, which can be applied to the detection of protein–protein, protein–DNA, and protein–RNA interactions (Swartzman et al., 2010; Hansen et al., 2014). Furthermore, the method was adapted for the identification of interactions dependent on post translational modifications. Recently, e.g., an SH2-PLA was established, which allows the quantification of interactions between an SH2 domain and phosphotyrosines in the EGFR using a microtiter plate format (Thompson et al., 2015). This method, which is highly sensitive and has a large dynamic range, has a wide array of applications both in basic and translational cancer research. Similarly, an *in situ* PLA variant was successfully applied to detect the Erα/Src/PI3K protein complex in breast cancer cells and patient samples (Poulard et al., 2014), suggesting that the method has the potential to be utilized as diagnostic tool.

Although several studies have generated quantitative information about PPIs using PLAs, e.g., through secondary methods like color segmentation image analysis (Gajadhar and Guha, 2010; Leuchowius et al., 2010; Pacchiana et al., 2014), the currently available variants cannot be utilized to define binding strengths of interactions. To obtain such information, knowledge about the abundance of both bound and unbound protein molecules would be required. However, PLAs remain powerful tools to validate interactions initially identified in high-throughput screens under physiological conditions.

## Fret-Based Methods

The fundamental theory of FRET was established in the first half of the 20th century (Cario and Franck, 1922). Its great potential for biological research, however, has only been realized in the past 20 years, after different techniques had been developed that allowed the application of FRET in biological systems (Mills et al., 2003; Wallrabe and Periasamy, 2005; Ma et al., 2014). This, in particular, includes the combination of FRET with microscopy techniques, which allow the investigation of PPIs with temporal and spatial resolution *in vivo* (Sun et al., 2013). FRET is a distance-dependent process in which, through dipole–dipole interactions, an exited fluorophore molecule (the donor) transfers energy non-radiatively to another fluorophore molecule (the acceptor), resulting in acceptor emission (Lakowicz, 2013). Alternatively, dark quenchers can be used as acceptors for studying, e.g., membrane–protein interactions (Cho et al., 2016). There are three main conditions that need to be met for efficient FRET: (i) there must be “spectral overlap” of the donor’s emission and the acceptor’s excitation spectra, (ii) the donor and acceptor fluorophores (termed FRET pair) must be in close proximity and (iii) the dipoles of the donor and acceptor must be aligned (Lakowicz, 2013). Due to the fact that FRET efficiency is proportional to the inverse of the sixth power of the distance between the donor and the acceptor, only fluorophores that are in very close proximity (<10 nm) show FRET (Clegg, 1995). Thus, FRET allows the detection of direct interactions between proteins, whereas methods such as FCCS, PLA, DULIP, or LUMIER with BACON cannot distinguish between proteins that directly interact or are only present in the same complex (Li et al., 2015).

To measure FRET with microscopic techniques several basic approaches have been developed. This includes acceptor photobleaching (Szabà et al., 1992), fluorescence life-time imaging microscopy (Wallrabe and Periasamy, 2005), spectral imaging (Chen, 2011), and sensitized emission, which still is the most commonly applied FRET method. Sensitized emission measurements can be performed using standard confocal and wide-field microscopes with appropriate filters or fluorescence microplate readers. Three channels are normally required for the imaging of donor, acceptor and FRET signals. The sensitized emission method, also called three-cube FRET, is based on the detection of acceptor fluorescence after donor excitation (Gordon et al., 1998; Mattheyses and Marcus, 2015). However, it is important to note that usually it is not possible to visualize sensitized emission directly due to contamination of the FRET signal by both donor and direct acceptor fluorescence. Thus, the measurement has to be corrected for donor bleed-through and acceptor cross-excitation, which can be performed through the calculation of calibration factors obtained from measurements with reference samples containing either donor or acceptor molecules alone (Mattheyses and Marcus, 2015). Currently, various algorithms are available to correct for these fluorescence contaminations, which all give comparable results (Zal and Gascoigne, 2004; Chen et al., 2006). Subsequent normalization to the donor or acceptor protein level (or a combination of both) provides a quantitative FRET signal (Hoppe et al., 2002; Zal and Gascoigne, 2004; Chen et al., 2006; Elder et al., 2009).

To study PPIs with FRET, the proteins of interest need to be tagged with appropriate donor and acceptor fluorophores. This is possible through the production of genetically encoded fusions with fluorescent protein tags in cells using multiple expression plasmids (Hochreiter et al., 2015). This includes FRET pairs such as ECFP/EYFP (**Figure 1E**), mTurquoise/mCitrine or EGFP/mCherry that are commonly applied for the investigation of PPIs in cells (Day and Davidson, 2012; Mattheyses and Marcus, 2015). A major strength of FRET-based interaction studies in living cells is that quantitative information about PPIs can be obtained. This is achieved through saturation experiments in which FRET is monitored in cells coexpressing a constant amount of donor-tagged protein with increasing amounts of acceptor-tagged protein or *vice versa* (Carriba et al., 2008; Martínez-Muñoz et al., 2014). Through such an approach, FRET_50_ values can be calculated, which provide an indication about the binding strength of tagged interacting proteins. However, it needs to be noted that FRET measurements in living cells can provide information about binding affinities only when the absolute concentrations of investigated proteins are known. Such information, however, is generally not available without additional measurements in standard FRET-based PPI studies (Sun et al., 2013). Nevertheless, a recent study demonstrated that reliable *in vivo* binding affinities between the proteins glutathione (GSH) and glutathione-*S*-transferase (GST) can be obtained from FRET measurements in intact cells (Chen et al., 2015). Thus, FRET microscopy and spectroscopy are powerful techniques that can provide highly reliable information about the binding strengths of PPIs, even at subcellular resolution.

## Bret-Based Methods

Bioluminescence resonance energy transfer is a biophysical technique that, similar to FRET, can be readily applied for quantifying PPI strengths in living cells (Pfleger and Eidne, 2006). One distinction between the two methods is that FRET involves energy transfer between two fluorophores, one of which requires extrinsic excitation by a suitable light source, whereas BRET occurs after oxidation of a substrate (e.g., coelenterazine) through a luciferase enzyme (**Figure 1F**). Previous studies indicate that different luciferase enzymes such as *Renilla* luciferase (Rluc) or NanoLuc in combination with various fluorophores (e.g., EYFP) are suitable for in-cell BRET experiments and for the quantification of PPIs using BRET_50_ values (Hamdan et al., 2006; Szalai et al., 2014; Brown et al., 2015). The assembly of G protein-coupled receptors, e.g., was successfully studied in mammalian cells with the help of BRET (Stoddart et al., 2015). Furthermore, it was shown that a sequential BRET–FRET technique (termed SRET) is able to detect the interactions between three proteins *in vivo* (Carriba et al., 2008). Combined BRET and FRET methods are powerful tools to analyze the assembly of higher-order protein complexes and the effects of posttranslational modifications on PPIs. Recently, a BRET–FRET approach was applied to study the oligomerization of the proteins CCR5, CD4 and CXCR4, which are of critical importance for the infection of cells by HIV-1 (Martínez-Muñoz et al., 2014). Thus, novel fluorescence and luminescence-based methods allow the systematic quantitative analysis of protein complexes in cell models. They might be advanced for routine validation of PPIs identified in high-throughput screens with qualitative assays (Rolland et al., 2014).

## Luciferase-Based Co-Immunoprecipitation Methods

Co-immunoprecipitation (Co-IP) is commonly used to detect PPIs in protein extracts (Phizicky and Fields, 1995). However, identifying interactions with Co-IPs is laborious and time consuming, making the method unsuitable for systematic screening. To overcome these limitations, a luminescence-based Co-IP assay – termed LUMIER – was developed, which provides at least semi-quantitative PPI information and can be performed in microtiter plates (Barrios-Rodiles et al., 2005). Here, bait and prey proteins are co-produced as FLAG and *Renilla* fusions in mammalian cells and interactions are detected by luciferase enzymatic assays in co-immunoprecipitates. LUMIER has the advantage that large numbers of bait/prey pairs can be systematically tested for putative interactions under relatively well-defined assay conditions. The method was successfully applied for the generation of a dynamic PPI network for the TGF beta pathway (Barrios-Rodiles et al., 2005) as well as for the identification of inhibitors of the Wnt pathway (Miller et al., 2009), indicating that it is suitable for the elucidation of novel signaling pathway components with high confidence.

The original LUMIER assay has the disadvantage that the FLAG-tagged bait proteins cannot be quantified in co-immunoprecipitates, which may lead to false negative results in large-scale PPI screenings. To overcome this limitation, an improved version of the LUMIER assay was recently established (Taipale et al., 2012, 2014), which was termed LUMIER with bait control (LUMIER with BACON). Here, the immunoprecipitated FLAG-tagged bait proteins are systematically quantified by ELISA. LUMIER with BACON, which can also be performed in microtiter plates, facilitates the calculation of quantitative interaction scores that can be used for hierarchical clustering of PPIs and the prediction of potential functional modules. Applying LUMIER with BACON, a quantitative chaperone interaction network was generated that enabled the identification of regulators of cellular proteostasis (Taipale et al., 2014).

A dual luciferase reporter pull-down (DLR-PD) assay for the detection of PPIs in mammalian cells was also reported (Jia et al., 2011). In this assay, bait and prey proteins are co-produced in cells as firefly and *Renilla* luciferase fusions, respectively. In addition, the expressed bait protein harbors a HAVI-tag that is recognized and biotinylated by the co-produced biotin-protein ligase BirA. The DLR-PD assay was shown to successfully detect nuclear and cytoplasmic PPIs in HEK293 cell lysates, suggesting that the method can be applied for PPI screening. However, pull-down assays with beads are not easy to scale up for high-throughput applications. To overcome this limitation, most recently a DULIP assay was developed for interactome mapping in mammalian cells (Trepte et al., 2015). This method can be performed in 384-well microtiter plates and can be automated for large-scale interaction screens (**Figure 1G**).

In DULIP assays the bait and prey proteins are co-produced as *Renilla* and firefly luciferase fusions in mammalian cells, respectively. In addition, the expressed bait protein harbors a protein A (PA) tag (Li, 2010) that allows the co-precipitation of bait/prey complexes in microtiter plates. The successful expression of bait and prey fusion proteins as well as the success of bait/prey co-precipitation can be quantified using DULIP. This enables the calculation of quantitative, normalized interaction ratios for all tested protein pairs, which can be utilized to create quantitative PPI interaction maps. The method, e.g., was capable of detecting the effects of point mutations on the interaction strength of synaptic proteins (Trepte et al., 2015), suggesting that it might be suitable for more comprehensive investigations of the effects of disease-causing mutations on PPIs. Taken together, luminescence-based assays are powerful PPI detection methods that, in the future, might allow us to obtain quantitative information about interactions in large-scale systematic studies.

## Conclusions and Outlook

Resulting from multiple high-throughput PPI screening efforts with genetic and biochemical methods (Stelzl et al., 2005; Yu et al., 2008; Rolland et al., 2014), we currently possess large databases with unexplored interactions. Their further characterization requires quantitative experimental strategies that are easy to implement in laboratories and allow the identification of interactions at medium to high throughput in mammalian cells. Recent developments indicate that quantitative PPI information can be generated *in vivo* with methods such as FCCS, BRET, DULIP, or LUMIER with BACON (**Table 1**). This opens new avenues for interactomics researchers because the dynamics and strengths of PPIs can be assessed for the first time with these techniques. Also computational approaches to predict or filter PPIs relevant to a given question will profit enormously from direct prioritization of PPIs based on quantitative interaction data. It seems now possible to capture a broad range of high-, medium- and low-affinity interactions and to link this information to specific cellular processes. In the long run, this will enable us to describe the molecular principles of biological systems in more detail and to improve our understanding of disease processes. We suggest that truly quantitative interactome research is now within reach and efforts need to be intensified to obtain comprehensive quantitative PPI data sets in living cells.

## Author Contributions

AB, PT, KK, and EW wrote the initial manuscript. AB, PT, KK, SS, and EW revised the manuscript and approved the final version.

## Conflict of Interest Statement

The authors declare that the research was conducted in the absence of any commercial or financial relationships that could be construed as a potential conflict of interest.
